# Does climate action bring peace? Assessing the geopolitics of renewables using global investment data

**DOI:** 10.1038/s44168-023-00045-6

**Published:** 2023-07-10

**Authors:** Juergen Braunstein, Andreas C. Goldthau, Konstantin Veit

**Affiliations:** 1 Institute for Economic Geography and GIScience, Vienna University of Economics and Business (WU), Vienna, Austria; 2 Franz Haniel Professor of Public Policy, Willy Brandt School of Public Policy at the University of Erfurt, Erfurt, Germany; 3 Geopolitics of Transitions in Energy and Industry, Research Group Lead, Research Institute for Sustainability - Helmholtz Center Potsdam, Potsdam, Germany; 4 Accenture Sustainability Services, Berlin, Germany

**Keywords:** Environmental social sciences, Social sciences

## Abstract

The transition toward renewables is central to climate action. The paper empirically tests whether renewables also enhance international peace, a hypothesis discussed in the International Political Economy (IPE) of renewables literature. It develops and tests hypotheses about the pacifying effects of renewables, with a view to establishing the foundations for analyzing more detailed causal mechanisms. These mechanisms rest on the ‘energy democracy’ debate, suggesting that a low carbon world sees less interstate tension thanks to more states being democratic; the ‘capitalist peace’ theorem, establishing that the deployment of renewables brings about economic development, reducing conflict; and the human security literature, positing that renewables reduce local-level reduce vulnerabilities, thus enhancing social stability and reducing violence. Using a longitudinal dataset on global renewable energy investment, econometric tests suggest that distributed renewable energy systems do not seem to foster democratic rule, nor do they have a significant influence on human development. Countering the energy democracy literature, it is a higher concentration of renewable investment that tends to increase stability/ absence of violence and human development, instead of decentralized investment patterns. We find no evidence for the ‘peace through prosperity’ argument. Overall, there is no support for the assumption that renewables bring about peace and reduce conflict. The paper critically discusses the limitations of these findings and suggests further avenues for empirical research.

## Introduction

The energy sector accounts for more than two-thirds of global greenhouse gas emissions^1^. The transition toward renewables is therefore central to climate action. Shifting to carbon-neutral energy sources may shape international politics no less than previous energy transitions did in past centuries. The fossil energy system has famously given rise to severe political and socio-economic pathologies. Resource rents tend to facilitate the capturing of democratic institutions, stimulate corruption and aggravate domestic tensions^2–4^. Fossil fuels may also trigger outright armed domestic conflict^5^. Because they are found only in a few select places in the world, states might fight each other over access to resources^6^, and even if unintended, there exist various pathways in which oil can lead to or fuel war^7^.

It is hoped that the low-carbon energy shift will change these patterns. In contrast to fossil fuels which are characterized by an uneven geographical distribution of global reserves, renewable energy is abundant across regions and countries. Domestically produced energy from wind or solar can therefore strengthen the autonomy of states and lower costly energy imports, thus reducing both economic and supply vulnerability^8^. Tensions over fossil-fuel resources may ease, as the latter becomes less central to countries’ economies^9^. Enhanced access to clean energy services may help to be the solution to domestic instability and conflict^10^. In sum, renewable energy may do away with many of the conflictual patterns characterizing the fossil fuel-based energy system^11^.

Renewables come with their own geopolitical pathologies^12^. Petrostates may find their income wither away^13,14^, fossil fuel assets may become stranded^15^, and competition may emerge around rare earth elements needed to manufacture renewable appliances^16^. It has also been argued that the transition can be uneven across countries, leaving some countries behind^17^. Clean technologies have been argued to bring about a new type of extractivism^18^ and exploitation^19^. Still, the global transformation from fossil to renewable energy brings about a systemic shift that is argued to address the root causes of fossil-induced conflict: less potential for political abuse thanks to geographically leveled availability of resources; energy abundance instead of collusion-induced scarcity, such as in oil; and lower central control of revenue flows thanks the decentralized nature of renewables, reducing the ability to mobilize resources for war^11,20,21^.

The present paper sets out to empirically test the assertion that renewables bring peace. This is by no means a trivial exercise. The first challenge relates to the fact that the emerging geopolitics of renewables debate tends to conflate the notions of peace with conflict or violence. Surely, the meaning of peace is not well conceptualized in the pertinent literature either^22,23^. Yet, peace is more than a mere “absence of violence”^24^; and reducing conflict comes with fundamentally different mechanisms and logics than enabling peace. Types of conflict range from armed to ethnic to outright civil war^25^. Moreover, it has been argued that human security and sustainable development^26^ but also democratic governance and participation^27^ are important aspects for reducing conflict. A robust research strategy, therefore, needs to theorize how the low-carbon energy transition and the deployment of renewables may effectuate more or less conflict, stability, violence, and peace.

Another challenge is empirical. In essence, the assertion that renewables help peace is hard to test, as much of the assumed impact lies in the future. Despite renewables claiming an increasing share in the energy mix, it will arguably take time until fuel-importing states lower their vulnerability thanks to homegrown energy production, until petrostates overcome their resource curse by developing alternative low-carbon business models, and until pathways from oil to war are broken for good.

Against this backdrop, the research strategy of the present paper centers on building on three select strands of literature in the political economy of conflict domain—the role of democracy, development and human security—to establish and assess distinct hypotheses. The first relates on an emerging debate on ‘energy democracy’, which establishes that thanks to their decentralized nature renewables provide a bottom-up push for democracy and participation. Renewables changing the political economy of domestic power distribution, in turn, fosters democratic peace on the international level. A second hypothesis asserts that the deployment of renewables improves national welfare and prosperity and alters actors’ incentives to engage in costly conflict. This results in what the literature has coined ‘capitalist peace’ on the international level. A third hypothesis rests on the human security literature and poses that decentralized renewables advance local and community-level economic development and reduce vulnerabilities arising from energy poverty. This results in higher social stability and reduced violence, with positive spillovers on international conflict levels.

In all three instances, contexts matter. It makes a difference whether renewables are deployed in a conflict-prone setting or a politically stable environment. The effects of introducing decentralized energy systems in a separatist region or an established and stable participatory democracy will arguably be different, as are the ways in which renewables and their material structures shape the incentives of involved actors toward more or less conflictive patterns of interaction. In short, the phenomenon of war and peace is characterized by equifinality. This, on the one hand, calls for specifying the scope conditions and the reach of the theoretical predictions. We do this by discussing the different assumptions underpinning each of the theories we build on, for conflictual and non-conflictual environments. We also specifically test for the interaction of conflict settings with renewable energy investment, so as to empirically distinguish between conflictual and non-conflictual contexts.

The hypotheses about renewables and peace are tested using econometric regressions in R. The data on renewables stem from the Thompson Reuters SDC Platinum database, which offers comparable longitudinal panel data on project finance across the globe. Data on development are from the World Bank, democracy is measured by the Varieties of Democracy data, and human security indicators include the UNDP’s HDI index as well as the World Bank’s Governance Indicators. We also include a battery of control variables in the empirical tests and run several subsets to further check for the robustness of our findings. The empirical results are sobering. Distributed renewable energy systems do not seem to foster democratic rule nationally, nor do they have a significant influence on human development. Levels of political stability correlate with decentralized energy supply but it is a higher concentration of renewable investment that tends to increase stability/ absence of violence. We do find some effect for the ‘peace through prosperity’ argument. Overall, however, the data suggests that there is little support for the assumption that renewables bring about peace and reduce conflict.

Before we proceed, two caveats are in order. First, the paper establishes distinct causal chains, whose empirical validity it assesses using a large-n approach but which it cannot test in detail. Rather, the statistical tests provide insights into whether renewable investment correlates with the selected indicators. Also, the paper cannot and does not claim that these are the only possible pathways. The call here is on a further empirical investigation of the effects of renewables on peace, notably using qualitative case studies, also with a view to identifying alternative causal chains.

Second, although the energy transition is gaining speed, the share of renewables in the energy mix is increasing only slowly. The present paper is therefore confined to only capturing the early indicators of the hypothesized effects renewables may eventually have on democracy, development and human security. That said, if said effects exist, our econometric strategy should be able to detect them even if remaining at a low level.

The next section “Three causal mechanisms: energy democracy, economic development and human security” elaborates on three distinct causal mechanisms by which renewables may lead to peace, zooming in on energy democracy, economic development and human security. In order to justify further investigation into the causal mechanisms whereby energy transitions might have a pacifying effect, we develop hypotheses on the relationship between renewables and peace. Our statistical analysis refines the research question from whether renewables bring peace in general to the issue of equifinality, that is of different causes possibly producing the same outcome in different contexts). The “Methods” section describes the data and specifies the econometric model. The “Results” section presents the results and the “Discussion” section discusses the findings against the main assumptions. Finally, we conclude and offer some thoughts on further avenues for academic inquiry.

## Three causal mechanisms: energy democracy, economic development and human security

### Energy democracy

Democracy is widely conceptualized as being inversely related to conflict. On the national level, democratic civil peace is found as more durable than autocratic peace^28^. On the international level, the democratic peace theorem links countries’ governance regimes to the likelihood of military conflict^29,30^. According to the ‘democratic peace theorem’, essentially the attributes of political systems determine which states will and will not go to war with each other (Elman, 1997). War is avoided because democratic decision-makers follow the norms of conflict resolution that characterize their own domestic political processes (Gowa 1995). If democracies go to war, they only do so if they stand a chance to win^31^. Furthermore, democratic structures curtail the discretionary behavior of elected leaders. Public debate and opposition slow down policy processes and leaders cannot act quickly, which cautions foreign policy behavior and reduces the likelihood of war (Herman and Kegley 1995). As a consequence, elected leaders cannot easily commit the state to war, which means that the political structures characteristic of democracies are principally geared against war^32,33^. What is more, if composed of democracies, international institutions are found to better performing when it comes to overcoming escalation pathways and prevent conflicts from resulting in war^34^.

An emerging debate on ‘energy democracy’ links renewables to political rule (for a genesis see Van Veelen and van der Horst^35^). Works mainly center on the effect of renewable energy on political and economic power^36^. As renewables tend to be decentralized and distributed in nature, as opposed to incumbent centralized fossil systems, they are argued to redistribute power to local communities and individuals^37^. Thanks to revenues from decentralized renewable energy predominantly resting with local actors, not big corporations and governments, citizens see empowerment and opportunity for voicing their preferences in the political process^37^. Distributed systems are argued to enhance citizen ownership of the energy network^38^, bring in non-traditional actors such as cooperatives, and empower all of the above^39^. At the same time, thanks to their ability to produce energy independently from the centralized grid, peripheral players may be emboldened to demand more political participation. Participatory governance emerges as a key element in giving ‘prosumers’ agency in the transition process and in shaping it^40^. In addition to seeing opportunity, energy prosumers may also have a material incentive in influencing the very process determining the economic environment they operate in. This increases accountability and governance^41^. Against the backdrop of the democratic peace argument, renewables therefore become a driving force for external state behavior: they may well have a democratizing effect on such states that deploy low-carbon energy systems. By extension, these states can be expected to be less likely to go to war. We refer to this as the ‘energy democracy peace hypothesis’.

The causal chain can, in sum, be described as follows: Renewables work to enhance distributive justice locally and foster democratic rule nationally. On the international level, a low-carbon world will see less armed interstate tension thanks to more states being democratic. Democratic peace ensues thanks to renewables changing the political economy of domestic power distribution. The specific hypothesis following this causal mechanism can be stated as follows:

*H1: Increasingly distributed energy systems enhance participatory democracy*.

That said, participatory democracy is a necessary but not sufficient condition for peace: it only represents an intervening variable, materializing through increasingly distributed energy systems. Yet, the interplay of decentralized energy systems, participatory democracy and peace is complex and may vary across peaceful and conflictive settings. For example, distributed energy systems may empower pre-existing separatist movements as they see increased material independence. By contrast, introducing distributed energy systems into stable and peaceful settings may create positive feedback contributing to more stability and peace.

### Economic development

Economic development has been identified as a key factor in understanding peace and conflict. At the most basic level, poverty gives rise to threats, including civil conflict^42^. Economic development, by contrast, is argued to reduce conflict, as “development retards war”^43^. Generally, a capitalist mode of production underpinning the national development model is said to temper the appetite for conflictual behavior of states, internally and externally, a debate known as the ‘capitalist peace theory’ (for a discussion, see ref. ^44^). The underpinning reasoning here ties into the broader tradition of ‘commercial liberalism’^45^, in which economically open states are found to pay more attention to the interests of important economic actors at home. In their foreign policy, they also tend to rely more on the resources and capabilities stemming from economic growth, rather than on military intervention^46^. Moreover, the opportunity costs associated with mutually beneficial economic activity such as trade discourage (interstate) violence. War or conflict is considered a product of failed bargaining due to incompatible interests. Peace results from involved actors not finding it worth pursuing costly conflicts over their differences^47^.

There exist various ways in which economic development may decrease the level and likelihood of external conflict. For ‘commercial liberalism’ to exert a pacifying impact, the level of development seems to matter. One is through economic openness which is found to increase average income and, by extension, enhance political stability and reduce conflict^48^. Development may break what Collier et al.^43^ call the ‘conflict trap’, setting in motion a virtuous cycle of economically prosperous countries becoming safer, fostering further subsequent development. Economic development may be particularly helpful in countries facing a high risk of civil conflict. Moreover, an important element highlighted by Mousseau^49^ is the link between economic development and impersonal contracting underpinning capitalist societies. Higher development levels are argued to bring about both higher dependency on markets and increased impersonal market transactions, which in turn necessitates third-party enforcement and dampens the appetite for conflict or violence in those transactions.

It has been argued that the low-carbon energy transition may open up new development pathways, particularly for nations in the Global South^50^. For instance, and in line with the rationales underpinning the Sustainable Development Goals, the deployment of renewables is argued to “boost job creation and economic growth”^11^, 33). Some see a wave of green industrialization looming for developing nations^51^, putting them on a sustainable and inclusive growth path. Renewables may thus help increase equality and prosperity because the ensuing economic benefits tend to be more widely distributed than in contexts of centralized energy systems that promote the concentration of financial benefits in the hands of corporations and governments. As a corollary, renewables may also offer alternative development pathways for resource-rich economies. Large natural resource endowments can undermine growth, distort the economy, and impede the development of domestic industry^52,53^. Renewables may allow diversifying national economies and shifting from fossil energy exports to the production of clean energy sources^54^. Finally, it has been suggested that renewables lead to more interdependence in financial relations, especially through cross-border infrastructure investment associated with renewables^55^. More financial interdependence via FDI in renewables makes it harder for states to inflict hardship on one economy without also experiencing harm themselves.

An economic development argument around renewables, prosperity and peace is similar to the energy democracy argument. Again, the analytical focal point rests on a changing political economy of domestic power distribution, as well as on changing rationales of involved—and possibly conflict-prone—actors. Yet, the assumed channels, whereby renewables become effective, differ. The causal chain can, in sum, be described as follows: renewable energy fosters economic development, which alters the incentives for domestic actors to engage in costly conflict. This, in turn, lowers the level and likelihood of conflictual behavior, domestically and by extension also internationally. The specific hypothesis following from this causal mechanism can be stated as follows:

*H2: Increasing deployment of renewables brings about economic development*.

Again, it is important to highlight the potentially moderating role of conflict settings in this relationship. Previous research suggests that armed conflict reduces foreign direct investment in the energy sector^56^ because it disrupts production and causes uncertainty. Blair and Christensen^57^ find that the degree to which investment is affected also depends on the geographical proximity to the conflict site. We would therefore expect to see a difference in the effect of increasing deployment of renewables on economic development between conflict-prone and peaceful settings, due to factors such as political risk and market conditions.

### Human security

Human security places individuals at the center of concern. To be sure, there barely exists an exact definition of human security^58^, and the literature offers both encompassing and narrow conceptions of the term^59^. What seems to be a consensus among scholars is the notion of human security representing “a shift of attention from a state-centered to a people-centered approach to security”^60^, 5). With this, the analytical focal point rests on threats to individual security, and on sources of individual insecurity. These can be manifold: hunger, political violence, climate change or poverty.

Conceptually, two main elements come together in human security. An important first element is direct threats to individual security and physical harm, notably through violence^61^. Human security implies the absence of organized violence, violence from state bodies, or from other domestic actors or sources. A second element lies in the individual ability to make life choices, which are seen as being directly linked to economic opportunity and human development. This link is captured by Amartya Sen’s seminal notion of “development as freedom”^62^. It is along those lines that the UNDP’s 1994 Human Development Report defined human security as entailing two components: “freedom from fear and freedom from want”^63^, 24). This means human security lies in the absence of threats to personal life or the community, as well as protection from threats against peoples’ economic base.

Decreasing levels of human security can lead to spillover to adjacent communities or neighboring nations and undermine their political or social stability. As argued by MacFarlane and Khong^61^, the UN has recognized that groups or regimes which systematically violate human rights at home are also likely to be threats to international security (229). Therefore, enhancing human security—both in the sense of individual security and in terms of human development—is susceptible to enhancing international stability and reducing conflict.

With this, the present paper subscribes to a broader notion of human security which proponents have argued allows understanding both the root causes of conflict and the policies to resolving it^60^. This is not to dispute the fact that the specific concepts underpinning the broader concept of human security are distinct and also tie into somewhat separate academic conversations^64^. The point here is to make use of a holistic conception of human security to establish how the low-carbon energy transition helps reduce human vulnerability and exposure to threats, hence reducing tensions and conflict.

Energy has been linked to various elements in the human security realm. For instance, energy poverty increases vulnerability and creates new inequalities^65^. Moreover, environmental *scarcities* have been found to give rise to violence under certain circumstances^66^. Access to modern energy services—that is, primarily, renewables—is believed to be crucial for ending energy poverty^67^. Renewables open up new economic opportunities and reduce inequality^68^ and foster human development^69^. Community-owned renewable energy may have a role to play in reducing environmental degradation^70^, and renewables are also expected to enhance community resilience against climate-induced threats^71^, which fosters social stability and reduces inter-communal conflict. There also exist numerous local socio-economic benefits^70^ related to the deployment of renewables, which has been shown to particularly benefit village-level prosperity^72^. Moreover, access to clean energy services is also said to empower females^73^ and to address the gender dimension of human security. Finally, international agencies and NGOs have started to promote renewables as a means of peacebuilding in conflict-affected regions, thus linking local conflict and the challenge of scarce energy resources^74,75^.

The causal chain can, in sum, be described as follows: renewables enhance human security by way of advancing local and community-level economic development and reducing vulnerabilities arising from energy poverty. This brings about social stability and reduces violence. Lower domestic potential for violence and enhanced economic opportunity may have second-order effects on international conflict thanks to positive spillovers. The hypotheses following from this causal mechanism can be stated as follows:

*H3: Distributed energy systems help human development*.

*H4: Distributed energy systems reduce violence and enhance social and political stability*.

In terms of contextualizing the above causal chains for human security, H3 and H4 address similar material incentive structures created by renewables as do the hypotheses derived from the theories of democratic peace and capitalist peace, albeit at a different analytical level. The interplay of distributed energy systems, human development and peace/conflict may therefore again vary depending on the moderating effect of conflict settings. Whereas some advocacy reports praise the opportunities offered by distributed RES for conflict-affected areas^76^, there are also concerns that pre-existing local conflict can complicate the deployment of renewable energy projects^77^. Either way, we may see a difference in the hypothesized effect of distributed energy systems on human security between conflictive and peaceful countries.

Table 1 summarizes the three causal mechanisms, ordered by level of analysis, and details the analytical notions underpinning the analysis.

**Table 1:** Theoretical concepts and causal mechanisms.

Independent variable	Causal mechanism	Dependent variable	Definition of “peace”	Theoretical concept
Total renewable investment	Renewables bring economic development and prosperity	GDP per capita	Absence of internal or external conflict	Capitalist peace
Distributed renewable investment	Renewables foster energy democracy	Democracy	Absence of (interstate) war	Democratic peace
Distributed renewable investment	Renewables increase individual economic empowerment and reduce vulnerability	Human development; stability/violence	Absence of domestic/local violence & conflict	Human security

## Methods

### Independent variables: project finance investment in renewable energy

In order to measure the effect of increasingly *distributed* renewable energy systems (H1, H3, H4), and the effect of increasing deployment of renewables *in total* (H2), we operationalize renewables as two independent variables, both based on project-level observations. The first is the *sum* of all RES project-finance investment into the energy sector of a given country, which allows capturing the aggregate level of renewable deployment in that country. The second is a measure of *concentration* of the project-level size of investment per country (as opposed to *total* investment per country), which tells us the degree to which renewable energy projects are decentralized or concentrated.

Though renewable energy investment may come in various shapes, project finance stands out in terms of the size of the investment volumes and in terms of growth. It outperforms other forms of renewable energy investment and has seen strong growth rates since 2004. By 2015, more than half of the total RES investment was through project finance^78^. Over the last decade project finance investment accounted for roughly 40–50 % of the total renewable investment volume^79^.

Project finance is a type of finance used for infrastructure or industrial projects with non-recourse or limited recourse financial structure. Project-level RES investment comprises both domestic and foreign sources of investment. The debt or equity invested is paid back from the cash flow generated by the project. Funding can flow from both public and private actors.

What is more, project finance comes with specific properties which make it a particularly potent financing instrument for renewable energy. Although project finance is typically used for investments in high-risk environments, it becomes increasingly important for financing renewable energy projects in developed states as well, particularly when it comes to smaller businesses and community energy projects^80^. The reason is that its financing structure allows developers low on equity capital to realize projects. Project finance can therefore play a crucial role “where large incumbent players have a high cost of capital, and small entrants do not (yet) have the size of balance sheets required for major investments” [80, p.20]. Consequently, project finance allows for different ownership types (community-owned, corporation-owned, state-owned, or PE-owned). As the present paper is based on the assumption that a distributed energy system rests on rather small projects, project-level finance is the more applicable investment type for our analysis compared to on-balance-sheet investment data, which typically captures capital investment in developed countries in sectors other than energy and transport.

Our longitudinal dataset originates from the Thompson Reuters SDC Platinum database, a leveled data resource on project finance across the globe. The dataset consists of roughly 8,500 project-level observations and encompasses a time span from 1991 to 2019. The dataset allows inference on the historical development of investments in renewables, as well as on the distribution of these investments over time and across countries. Using the search function for *renewable energy, solar, wind, hydroelectric, geothermal* and *biomass*, the SDC Platinum database picked up 8493 discrete deals/announcements worldwide, with an estimated total value of US$2,890 bn. Triangulating this volume with other pertinent sources on RES investment, the SDC Platinum database seems to cover a representative share of all global investments. For instance, Bloomberg New Energy Finance estimates the total cumulative global green investment between 2010 and 2019 at US$2.7 trillion^81^. The estimated value of all deals/announcements worldwide in our dataset between 2010 and 2019 is US$1,88 bn, which amounts to a 69% share of the reference value from BNEF. Comparing our data with the IEA energy investment data at about the same time span^82^ yields a similar picture: our project finance dataset accounts for approximately two-thirds of total global renewable capacity investments.

**Fig. 1:**
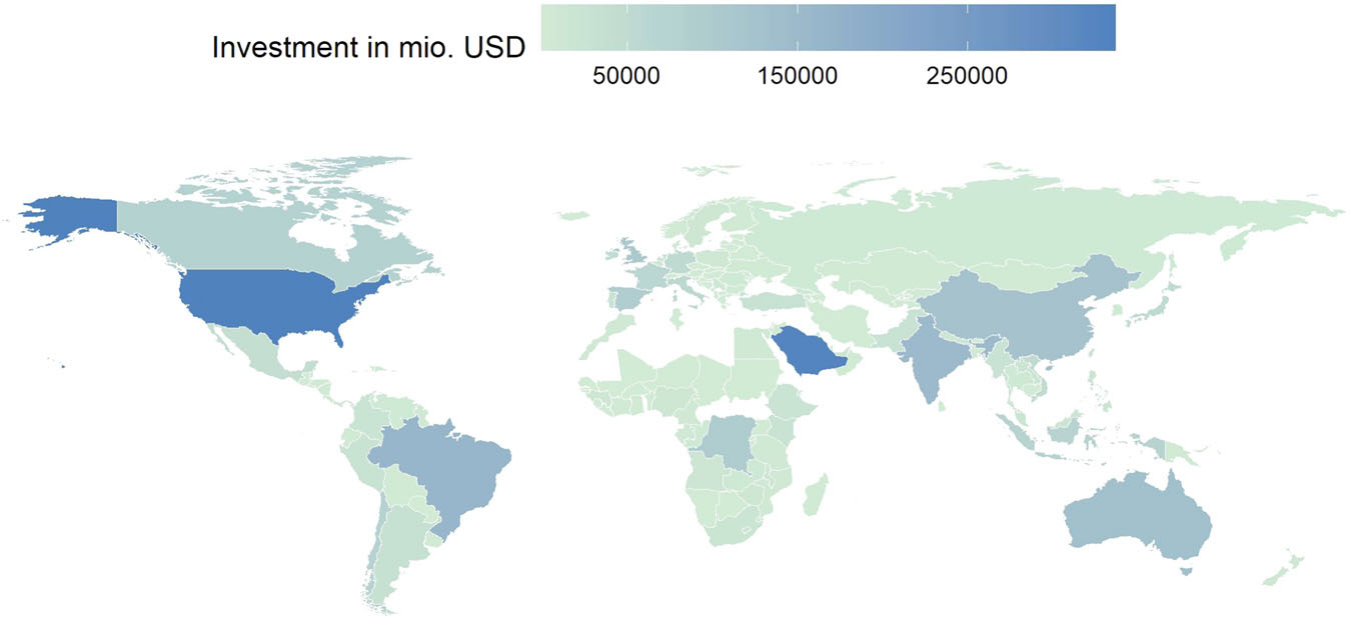
The United States, Saudi Arabia, Brazil, India, and Australia are the top 5 countries with the highest cumulative RE investment total over the time studied. Investment in renewable project finance per country (1996–2019).

Figure 1 depicts how renewable project finance investment is distributed globally.

Our first independent variable captures the effect of distributed renewable energy investment on democracy (H1), human development (H3) and political stability (H4). Reflecting the assumption that a decentralized energy system consists of projects with a rather small investment volume, we use the project cost variable in the dataset as a proxy to estimate the level of decentralization: the more small-scale investments, the more decentralized the investment in a given country—leading to, ceteris paribus, a more distributed energy system. Since we are interested in country-level effects, we aggregate all annual investments per country into a single index, thus creating a Herfindahl-Hirschman Index (HHI) for each country and year. The HHI is a measure that indicates the degree of concentration of all investments in renewables. By extension, it provides information on the level of decentralization of RES investment. We acknowledge there are shortcomings to using the HHI. For example, the HHI becomes small in case of a relatively high number of investments of similar size even though these investments may be very large. While the score suggests a decentralized energy system, the latter may in fact not have such a property. Still, the HHI is an adequate proxy variable to estimate the degree of decentralization of RE investments.

1 $${{\rm{HHI}}}_{{c},0}={\sum }_{({\rm{i}}=1)^{\rm{n}}}{\rm{S}}_{{({\rm{c}},{\rm{i}})}^{2}}$$

The Herfindahl-Hirschman Index is constructed by summing up the squared shares of every project investment value per total investment in a given country *c*, with n being the number of projects per country. We compute the HHI for each country and year. It is less sensitive toward outliers compared to a simple average of project investments. Furthermore, HHI is not invariant to the number of projects. This makes it a suitable indicator for our research since we want to depict concentration/ decentralization by taking into account the number and size of projects. While the existing literature frequently treats the nature of RES as decentralized as opposed to centralized conventional energy, our dataset allows disaggregating concentrated RES and decentralized RES.

The second independent variable is the total RES investment per capita in a given country and year. We use this variable to test the potential effect of increasing the deployment of renewables on economic development (H2). Complementing the concentration measure of RE investment with a variable capturing total investment allows accounting for the different levels of analysis of our hypotheses.

Our tests do not differentiate between different types of renewables. This is an analytical choice. To be sure, a particular type of renewables may come with its own contestations, as shown for instance by the discussion surrounding land use and the deployment of wind energy^83^. However, the focus of the present investigation is on the impact of renewables on material incentive structures and their potential effects on peace/conflict, an effect that goes beyond the short-term—at times also contested—nature of different renewable types, and which we control for by lagging our explanatory variable (see below). Also, we re-ran our tests with a sample excluding hydroelectricity projects, based on the intuition that these projects are underpinned by large investment volumes which may bias our concentration variable. As reported in Supplementary Table 2, the findings did not differ substantially from estimates including all RES types. Other large types of renewable energy projects, such as offshore wind or concentrated solar power, account for only 5% respective 1.5 percent, of the total number of projects in our dataset.

### Dependent variables

Democracy is measured using the *participatory democracy index* of the Varieties of Democracy (V-Dem) project. The index ranges from 0 to 1, with the latter representing ideal participatory democracy and the former representing the absence of democracy. The index therefore indicates the degree to which participatory democracy is achieved in a given country^84^. V-Dem data cover 202 countries for the time span relevant to the present paper, that is 1990 to 2019. The V-Dem index is particularly well-suited for the purpose of this paper because it emphasizes active participation by citizens in political processes, while other classic democracy indicators such as the Freedom House index focus solely on features of representative government. We also cross-checked the robustness of the statistical tests based on the V-Dem with Polity IV data^85^ as well as with other democracy indicators from V-Dem. Polity IV data vary less due to their discrete index and therefore are less responsive to changes in the independent variables, making the V-Dem indicator more suited for our analysis.

As for development, we use Worldbank data on *GDP per capita in current US$*, covering all years from 1960 until 2019^86^. The potential effect of RES investment on human development, the first dimension of human security, is measured using the classic Human Development Index^87^. HDI data are available from 1990 until 2018 and range from 0 to 1, with 1 representing the highest level of human development. The *political stability and absence of violence/terrorism* index of the well-established World Bank Governance Indicators^88^ represents the second dimension of human security. This index measures the likelihood a government is destabilized by unconstitutional or violent means—which can be argued to directly affect individual security. Country scores range from −2.5 to 2.5, with 2.5 representing the highest stability. It covers 21 years between 1996 to 2019.

### Control and interaction variables

In order to control for factors that might interfere with or influence the statistical results, we include a set of selected control variables. We focus on a small number of select control variables, to avoid strong multicollinearity and post-treatment bias. A case in point is good governance, which not only matters for renewable investment propensity^89^ but also influences all study variables. Our regressions therefore include two variables from the World Bank Governance Indicators:^88^ regulatory quality, measuring a government’s ability to provide adequate regulations that enable and promote private sector development; and the rule of law measure, which indicates the quality of contract enforcement, property rights and courts.

Moreover, financial market development is key for renewable energy investment^90,91^ but also likely to impact economic growth^92,93^, and other study variables. To control financial markets, we use the financial development index database of the IMF. The financial development index not only measures the private credit or stock market capitalization to GDP ratio but combines several indicators on the depth, accessibility, and efficiency of financial markets and institutions^94^.

Further, natural resource wealth and fossil fuels dominate exports and may impact a country’s economy and the degree to which available investment goes into renewable energy. We therefore account for resource wealth by controlling for the share of fuel exports in total merchandise exports of a given country, using the pertinent Worldbank indicator^95^. As a relative measure, fuel export shares indicate the importance of fossil fuels for a country’s economy, independent of its size.

Endowment with renewable or fossil fuel resources are relatively time-invariant and controlled for with a fixed effects regression analysis (see below). We therefore do not include controls for those, nor for any time-invariant factors which cause heterogeneity between the countries in the sample.

Finally, some of the dependent variables feature as controls in other hypotheses, as detailed in Table 2. Summary statistics for all variables are reported in Supplementary Table 1.

**Table 2:** Overview of control variables and hypotheses.

Control variable	H1: Democracy	H2: Economic development	H3: Human development	H4: Political stability
Rule of Law	X			
Regulatory quality		X	X	
Financial Development Index	X	X	X	X
Fuel exports as share of merchandise exports		X	X	X
Political stability and absence of violence	X	X	X	
GDP per capita	X			
HDI				X

In order to test for a potential interaction between our predictor variables of RES investment with conflict settings, we build a categorical variable with levels 0 and 1, indicating whether there was an armed conflict in a given country in a given year. We build this variable based on the UCDP/PRIO Armed Conflict Dataset, v20.1^96,97^. The dataset defines armed conflict as “a contested incompatibility that concerns government and/or territory where the use of armed force between two parties results in at least 25 battle-related deaths in a calendar year”^96^, p. 618f). We include intrastate and internationalized intrastate conflicts in the sample. For all conflicts in the dataset since 1991 (when our investment data started), the location of this conflict (a country name) is coded 1. The interaction is tested with a cross-product term of the respective investment variable with the conflict dummy.

To ensure comparability over time and across countries, we standardized all unbound variables, which is particularly relevant for fixed effects models.

### Econometric strategy

The econometric tests rest on a panel analysis with country and time fixed effects, controlling for every country, year, and demeaning values per country. (To demean or to center a variable in a panel dataset means that for each annual observation of country A the mean of country A is subtracted.) The advantage of panel data lies in the fact that they allow identifying causal effects under weaker assumptions (compared to cross-sectional data). The fixed effects analysis is executed in R using the package plm^98^. The fixed effects regression equation is specified as

2 $${Y}_{c,t}={{\rm{\beta }}}_{1}{{inv}}_{c,t-1}+{{\rm{\beta }}}_{2}{{\bf{control}}}_{c,t-1}+{{\rm{\mu }}}_{c}+{{\rm{\varepsilon }}}_{c,t}$$

with *Y* being the dependent variable of interest (democracy, economic development, political stability or human development), measured at time *t* in country *c*, and *inv* representing our measures of renewable energy investment (by concentration measure or total investment measure). *Control* is a vector of our controls for possible confounders.

Using country fixed effects, we remove time-constant, unobserved attributes of the units being studied while allowing them to be correlated with our explanatory variables. This hedges our model against time-invariant factors such as geography and prevents estimators from being biased because of omitted time-invariant characteristics^99^. Moreover, we ran F tests for individual and time effects which suggested fixed effects regression being preferable to a pooled OLS. Furthermore, the Breusch Pagan Lagrange Multiplier Test for time fixed effects revealed the necessity of using time fixed effects for the whole panel. Finally, the Breusch Pagan LM test of independence shows that our panel suffers from contemporaneous correlation across units, and our error term is heteroskedastic. Both are typical for time-series-cross-section data. We therefore use panel-corrected standard errors^100^ in all our regressions to account for cross-sectional heteroskedasticity and correlation. Tests were run to see if the data meets the assumption of collinearity, and they indicate that multicollinearity is not a concern (Variance Inflation Factor (VIF) < 8 in all models, <5 in all but one model, excluding models with interaction term). For the regression, we lag our explanatory variables to achieve quasi-exogeneity. We apply a 1-year lag, as widely done. Longer lags would be possible as well, but they require an additional theoretical justification so as to avoid arbitrariness. A replication of the tests with t-2 caused no change in the significance of the main effects, supporting our choice of a 1-year lag.

Although our investment data reaches back to 1991, we use data from 1996 onward, the first year when all relevant control variables were fully available. The data run through 2019, which is a time span of 24 years. Our panel is unbalanced. The reason for missing data points may lie in using a variety of data sources. However, there is no reason to assume a systematic correlation of missing data with the idiosyncratic error, which is why the unbalanced panel is of no concern for us^99^. All tables report the within R-squared, as it is common in fixed effect regressions^98^, e.g., we are interested in the explained variation after having adjusted for time and unit effects.

### Reporting summary

Further information on research design is available in the Nature Research Reporting Summary linked to this article.

## Results

### Effect on participatory democracy

The regression suggests that distributed renewable energy does not have an effect on participatory democracy (Table 3). Indeed, *RE inv. HHI* is a significant predictor for democracy in the bivariate model (column 1) and in model 2, controlling for GDP per capita; yet, the effect is the opposite of what we expected: on average, a one-unit increase in investment concentration across time *increases* the participatory democracy index by 0.01 points per country. As per the hypothesis, we expected *RE inv. HHI* to be a negative predictor for democracy levels, as a low concentration of renewable energy investment indicates a high level of decentralization.

**Table 3:** Influence on level of democracy.

	Participatory democracy index (Varieties of democracy)						
	(1)	(2)	(3)	(4)	(5)	(6)	(7)
RE inv. HHI	0.010^*^	0.011*	0.003	0.009	0.001	0.005	−0.002
	(0.006)	(0.006)	(0.006)	(0.006)	(0.006)	(0.005)	(0.005)
GDP per capita		−0.005^**^			−0.002		−0.002
		(0.002)			(0.002)		(0.002)
Rule of law			0.063^***^		0.067^***^		0.067^***^
			(0.010)		(0.011)		(0.011)
Pol.stab			0.010^**^		0.011^**^		0.011^*^
			(0.005)		(0.005)		(0.005)
Fin.dev.index				−0.101^***^	−0.140^***^		−0.142^***^
				(0.024)	(0.029)		(0.029)
Conflict						−0.033^*^	−0.018
						(0.018)	(0.015)
RE inv. HHI_conflict						0.024	0.019
						(0.016)	(0.014)
Observations	1053	1043	994	1026	968	1053	968
R^2^	0.004	0.006	0.065	0.012	0.082	0.012	0.087
F Statistic	3.235^*^ (df = 1; 883)	2.536^*^ (df = 2; 873)	19.112^***^ (df = 3; 826)	5.437^***^ (df = 2; 861)	14.339^***^ (df = 5; 804)	3.456^**^ (df = 3; 881)	10.985^***^ (df = 7; 802)

*p < 0.1; **p < 0.05; ***p < 0.01.

When including other control variables—governance and financial development—the effect of decentralized RES investment is no longer significant in any of the models (columns (3) and (4)). Instead, governance and financial development indicators become strongly significant predictors for *democracy* (column (5)). The R^2^ is generally low for all estimates, yet this is a rather typical pattern in fixed effects models^99^.

When testing for an interaction effect of *conflict* with *RE inv. HHI*, we do not find a significant pattern (columns (6), (7)). In sum, the concentration of RES investment is no significant predictor for democracy levels, neither in peaceful nor in conflict settings.

We replicate these models twice, substituting our democracy variable with two different democracy indices (Supplementary Table 3). The findings are essentially identical to those reported in Table 3.

### Effect on economic development

Turning to our second hypothesis, we do not find evidence to suggest that more investment into renewables brings about more economic development, as demonstrated by the fixed effects regression in Table 4. We expected *RE inv. total per capita* to be a positive predictor for economic development, measured by GDP per capita. We do not see a significant correlation in any of the models. Testing for the role of conflict settings, this finding does not change, either (column (5) and (7) In contrast, our set of control variables—governance, financial development, and share of fuel exports in total merchandise exports, shows strong predictive power in all models.

**Table 4:** Influence on level of economic development.

	Economic development (GDP per capita, Worldbank)						
	(1)	(2)	(3)	(4)	(5)	(6)	(7)
RE inv. total per capita	−0.013	−0.011	−0.010	−0.011	−0.012	−0.004	−0.001
		(0.013)	(0.013)	(0.014)	(0.015)	(0.013)	(0.015)
Regulatory quality		0.514^***^				0.448^***^	0.452^***^
		(0.119)				(0.119)	(0.119)
Pol.stab		0.262^***^				0.259^***^	0.274^***^
		(0.063)				(0.058)	(0.064)
Fin.dev.index			1.453^***^			1.287^***^	1.297^***^
			(0.517)			(0.472)	(0.473)
Fuel.exports.share				−0.011^***^		−0.012^***^	−0.012^***^
				(0.004)		(0.004)	(0.004)
Conflict					−0.081^*^		0.085
					(0.045)		(0.081)
RE inv. total_conflict					−0.002		−0.024
					(0.038)		(0.038)
Observations	1019	959	1002	946	1019	876	876
R^2^	0.001	0.079	0.018	0.015	0.002	0.102	0.103
F Statistic	0.744 (df = 1; 876)	23.496^***^ (df = 3; 818)	7.995^***^ (df = 2; 862)	6.233^***^ (df = 2; 808)	0.591 (df = 3; 874)	16.803^***^ (df = 5; 743)	12.166^***^ (df = 7; 741)

*p* < 0.1; *p* < 0.05; *p* < 0.01.

### Effect on human development

Turning to *Human Development*, we expected *RE inv. HHI* to be a negative predictor for HDI levels which, according to our hypothesis, would instead increase with increasing levels of decentralization in RES investment. The results of the fixed effects regression in Table 5 suggest the opposite. In the bivariate model (1), *RE inv. HHI* indeed is a significant predictor for *HDI*, yet a one-unit increase in investment concentration across time leads to an increase in the Human Development Index by 0.005 points on average for a given country. In other words, concentrated energy systems seem to correlate with economic empowerment.

**Table 5:** Influence on level of human development.

	Human Development Index (UNDP)						
	(1)	(2)	(3)	(4)	(5)	(6)	(7)
RE inv. HHI	0.005^**^	0.003	0.005^**^	0.005^**^	0.002	0.009^***^	0.006^***^
	(0.002)	(0.002)	(0.002)	(0.002)	(0.002)	(0.002)	(0.002)
Regulatory quality		0.011^***^			0.005^**^		0.006^**^
		(0.002)			(0.002)		(0.002)
Pol.stab		0.002			0.001		0.001
		(0.002)			(0.002)		(0.002)
Fin.dev.index			0.026^**^		0.056^***^		0.057^***^
			(0.013)		(0.014)		(0.013)
Fuel.exports. share				−0.0001	−0.0001		−0.0002
				(0.0001)	(0.0001)		(0.0001)
Conflict						0.016^***^	0.015^***^
						(0.005)	(0.005)
RE inv. HHI_conflict						−0.024^***^	−0.018^***^
						(0.006)	(0.005)
Observations	963	903	949	895	826	963	826
R^2^	0.008	0.026	0.014	0.010	0.045	0.045	0.075
F Statistic	6.720^***^ (df = 1; 799)	6.623^***^ (df = 3; 741)	5.625^***^ (df = 2; 788)	3.578^**^ (df = 2; 740)	6.438^***^ (df = 5; 677)	12.578^***^ (df = 3; 797)	7.867^***^ (df = 7; 675)

*p < 0.1; **p < 0.05; ***p < 0.01.

When including the control variables of governance, the effect of *RE inv. HHI* is no longer significant (column (2)). The effect of *RE inv. HHI* is significant in model (3) and (4). Any effect withers away when testing for all control variables at the same time (column (5)). *RE inv. HHI* is no longer a significant predictor for *HDI*. Instead, regulatory quality and financial development seem to be positive and significant predictors for HDI, with an R^2^ of 4.5%. Overall, the findings suggest that decentralized RES (investment) does not have an influence on levels of human development.

This picture changes when testing for the influence of conflict settings. In model (6), we find a significant interaction effect of *conflict* with *RE inv. HHI*. This suggests that the effect of higher investment concentration on HDI levels is weaker in conflict settings, compared to observations without conflict. These findings are robust against all control variables (column (7).

### Effect on political stability and absence of violence

Finally, the tests as depicted in Table 6 reject the hypothesis that distributed renewables enhance political stability and reduce violence, as captured by the *pol.stab* indicator. The results for the bivariate model suggest that a one-unit increment in the concentration of RES investment leads to an increment of the political stability and absence of violence indicator by 0.103 points on average. These results are robust but point to a causal relation that is inverse to our hypothesis, which predicted *RE inv. HHI* to be a negative predictor for *pol.stab*. When controlling for financial development, fuel exports share and human development, the significance level of *pol.stab* does not change in models (2) and (3). It decreases slightly when including only the HDI control. When including all control variables at the same time (5), *RE inv. HHI* is still significant on the 5% level, with an R^2^ at 2.1%. This implies that RES investment seems to matter for political stability and the absence of violence, yet it is a higher concentration of RES which seems to enhance political stability rather than decentralized RES systems. We do not test for our fourth hypothesis related to RES investment and conflict settings because the dependent variable itself includes a dimension of conflict.

**Table 6:** Influence on political stability and domestic violence.

	Political stability and absence of violence				
	(1)	(2)	(3)	(4)	(5)
RE inv. HHI	0.103^***^	0.108^***^	0.090^***^	0.087^**^	0.083^**^
	(0.035)	(0.037)	(0.035)	(0.036)	(0.036)
Fin.dev.index		0.648^**^			0.419
		(0.263)			(0.271)
Fuel.exports.share			−0.001		−0.001
			(0.002)		(0.002)
HDI				2.122^***^	1.647^**^
				(0.596)	(0.752)
Observations	1013	981	923	995	904
R^2^	0.010	0.020	0.008	0.022	0.021
F Statistic	8.743^***^ (df = 1; 841)	8.306^***^ (df = 2; 818)	3.237^**^ (df = 2; 767)	9.266^***^ (df = 2; 828)	3.958^***^ (df = 4; 751)

*p < 0.1; **p < 0.05; ***p < 0.01.

## Discussion

The statistical tests provide several important insights on the primary research question of the present paper. First, distributed energy systems can hardly be expected to enhance democracy, no matter whether there is conflict or not. In fact, the bivariate test suggests that the correlation is inverse: it is more *concentrated* RES investment that strengthens democracy. With this, a core assumption of the energy democracy literature is put in question, that is renewables redistributing power to local communities and individuals, and by extension foster democratic rule at the national level. To be sure, the democratizing effect of local-level RES deployment may still be small and unfolding primarily within local communities, given that renewables have not yet claimed the majority share in the energy mix. But even if only unfolding, the effect should be empirically visible.

As a corollary, the empirical findings do not support the ‘energy democracy peace hypothesis’. Because renewables are not found to have a democratizing effect on such states that deploy low-carbon energy systems, they can also not be expected to influence state behavior abroad. By extension, even if states deploy renewables at a large scale, the lack of an ‘energy democracy effect’ suggests they may not necessarily abstain from going to war thanks to embracing distributed energy systems at home. In short, renewables cannot be assumed to bring peace thanks to energy democracy.

Second, investment in renewables does not seem to significantly foster economic development. While the deployment of renewable energy projects might indeed bring about benefits for local communities, particularly in terms of energy access, this does not add up to traceable effects on GDP per capita. Our results show that financial market development, regulatory quality and political stability are strong predictors of economic growth, as confirmed by pertinent previous studies^101–103^. Renewable investment might well be a result of these factors and thereby its effect could be moderated through the control variables.

While increasing energy investment and thus supply is broadly associated with increasing prosperity and development^104^, singling out the effect of renewable energy deployment is difficult. The share of renewable projects within total energy investments was comparably small for most of the time span covered by our data. It is only since 2015 that renewable capacity growth accounts for more than 50% of global capacity additions (BNEF/UNEP 2020) while long-term investment trends in fossil fuels started to fall, with 2021 and 2022 being exceptions^105^. After some years of stagnation and even slow-down, RE investment started to accelerate strongly within the last 5 years^106^. (2020 to 2022 are not part of our analysis due to data limitations.)

Against this backdrop, the hypothesis based on the capitalist peace rationale needs to be rejected: as per the statistical test, renewables do not seem to induce development and therefore lower the level and likelihood of conflict. The relation between renewable energy investments and GDP per capita needs to be further analyzed. While we tested the hypothesis of renewable investment bringing about economic development, causality might also work the other way around (i.e., countries with high GDP per capita are better positioned to be early movers). One way of exploring this could be through case studies so as to develop typological theories identifying the specific conditions (e.g., GDP per capita level, unit costs of renewables, economic structure) under which RES are adopted.

Third, the findings call into question some of the assumptions underpinning the human development argument made by proponents of renewables. To be sure, renewables can truly transform the lives of low-income communities. They provide locally available clean energy, which power refrigerators, keeps the lights on for pupils studying at night and facilitate important innovations such as pay-as-you-go systems spread even into rural communities. But their effect on human development may overall remain modest, and a far cry from the prominent promise of renewable-induced prosperity. What is more, political stability and lower violence do not seem to be induced by distributed energy systems. Instead, we observe that more concentrated investment, that is, large-scale projects and, as a corollary, less ‘energy empowerment’ has a positive effect on political stability. This suggests that indeed the characteristics of an energy system do matter—albeit not in the way predicted. It also points to a less observed flip side of economic or political empowerment, which consists of it possibly also representing a driver of conflict.

Extending the analysis to account for the possible moderating influence of conflict settings reveals that context indeed matters. A case in point: the presence of an armed conflict reduces the effect of RES concentration on HDI. This is intuitive, as conflict hampers the potentially positive impact of RES on human development. Taken to the extreme, if the effect of concentration in conflict settings is not only smaller but even negative, this would imply that increased RES distribution (i.e., negative concentration) correlates with higher levels of human development. Our findings do not allow us to make this conclusion with certainty. They are robust, however, as regards the fact that the influence of distributed RES on human development is contingent on conflictive or peaceful settings.

The statistical results of the present paper call for further investigation. For one, the existing capital stock of the energy sector may make a difference: an identical volume of investment will arguably matter less in the US than it does in, say, sub-Saharan Africa. Further research could, for instance, explore the relative contribution of RES investment to conflict and peace, rather than looking at absolute measures. Moreover, the findings for energy democracy may point to alternative explanations. GDP per capita, governance and the level of financial development indeed correlate strongly with democracy. Yet, the underlying driver here may be one of the well-run countries attracting investment, as a corollary of which they also embrace democracy. What is more, it is not inconceivable that the effect we aimed at observing when testing for distributed energy investment—citizen empowerment—is at a scale that is not appropriately captured by the chosen data. Although V-Dem entails fine-grained data, it possibly still does not reflect the relevant micro-level effects. More disaggregated democracy data may deliver additional insights, as may longer time lags in the statistical tests. The call here is on complementary qualitative research to uncover the impact of renewables on empowerment at household and individual levels. Case studies on community energy and local participation processes can unravel hidden dynamics here. More generally, democracy and development are underpinned by long-term processes, and it takes time for these processes to manifest. Therefore, although renewables do not seem to have a pacifying effect via democracy or human security, they may be relevant for peace and stability through other mechanisms—or simply require more time. Finally, the statistical findings call for a more thorough qualitative analysis of the interaction effect between RES investment and conflict settings, to uncover the causal dynamics at work.

Several points of departure come to mind for a research agenda going forward. To start with, the theoretical assumptions underpinning the renewables-peace hypothesis may require further refinement. For example, while democratic structures may foster benign international behavior, democratic structures per se are not sufficient for peace. Public opinion set broad limits to foreign policy choices, and the democratic process can increase the chances of war especially if public opinion is not pacific^107^. The more fundamental question relates to criticism of democracy may simply be perceived as too ‘static’ to be analytically telling^108^. Moreover, the literature around environmental peacebuilding^109^ may offer promising starting points for theorizing on how addressing the possibly conflictual issues around renewables, such as land use, can foster cooperative behavior among local actors.

An absence of evidence famously is not evidence of absence. The fact that renewables are not found to positively impact conflict patterns does not mean it does not happen. Therefore, it will be important to develop a more process-oriented explanation of the possible way in which renewables relate to conflict or peace. Using deviant cases may be useful to that end and to help more robust theory building^110^. The use of temporal before and after research designs (i.e., using the introduction of renewables as a threshold) combined with process tracing may also allow refining some of the hypotheses, especially the human security argument, developed in this paper. It is hoped that the present paper offers inspiration for in-depth, qualitative empirical and theoretical analysis along these lines.

Renewables are central to climate action. Yet, the results of the present paper suggest that there is little reason to assume that renewables may also help peace. Clearly, improving the lives of people through decentralized energy systems is a stand-alone cause. Encouraging renewable energy investment for the purpose of lowering conflict levels between communities or countries, by contrast, arguably is ill-targeted. Linking renewables to more benevolent geopolitics may, therefore, be an interesting intellectual exercise. In policy terms, however, this paper suggests caution.

### Supplementary information

Supplementary Information

Reporting Summary

## Data Availability

Due to its proprietary nature, and since the material is owned by a for-profit company, supporting data from the Thompson Reuters SDC Platinum database cannot be made openly available. Further information about the data and conditions for access are available on the following website: https://www.refinitiv.com/en/products/sdc-platinum-financial-securities. Data derived from V-Dem, Polity IV, the Worldbank, the IMF as well as UCDP/PRIO Armed Conflict Dataset and v20.1 are publicly available.
