# Supplementary material for: Does climate action bring peace? Assessing the geopolitics of renewables using global investment data
Source: NPJ Clim Action. 2023 Jul 10;2(1):14. doi: 10.1038/s44168-023-00045-6 (PMC11062295; doi:10.1038/s44168-023-00045-6)
Supplement: Supplementary file 1 — Supplementary Information [file 44168_2023_45_MOESM1_ESM.docx]

# Supplementary information

## Supplementary Table 1: Statistical Summary

| **Statistic** | **N** | **Mean** | **St. Dev.** | **Min** | **Pctl(25)** | **Pctl(75)** | **Max** |
| --- | --- | --- | --- | --- | --- | --- | --- |
|  | | | | | | | |
| VDem_libdem | 3,495 | 0.431 | 0.265 | 0.018 | 0.178 | 0.676 | 0.887 |
| VDem_partipdem | 3,502 | 0.357 | 0.205 | 0.014 | 0.171 | 0.549 | 0.808 |
| PolityIV_polity2 | 3,210 | 4.098 | 6.216 | -10.000 | -1.000 | 9.000 | 10.000 |
| gdp_cap | 3,603 | 11,898.140 | 17,108.730 | 102.598 | 1,016.854 | 15,530.310 | 102,913.500 |
| HDI | 3,290 | 0.671 | 0.169 | 0.236 | 0.533 | 0.807 | 0.954 |
| Political violence | 3,192 | -0.083 | 0.917 | -2.845 | -0.714 | 0.678 | 1.760 |
| RE Inv. HHI | 1,132 | 0.680 | 0.337 | 0.032 | 0.363 | 1.000 | 1.000 |
| RE Inv. total | 1,132 | 2,524.612 | 8,545.590 | 0.700 | 153.800 | 2,059.950 | 200,000.000 |
| RE Inv. Meaned | 1,132 | 854.944 | 6,682.396 | 0.700 | 90.050 | 515.496 | 200,000.000 |
| Total RE capacity | 2,870 | 9,406.717 | 35,883.040 | 0.000 | 173.675 | 4,810.925 | 758,626.400 |
| Fuel exports share | 2,929 | 15.495 | 24.751 | 0.000 | 1.073 | 15.875 | 99.656 |
| Education | 3,290 | 0.604 | 0.189 | 0.101 | 0.460 | 0.752 | 0.946 |
| RISE score | 952 | 43.110 | 21.014 | 0.000 | 27.335 | 58.666 | 96.554 |
| Financial developm. index | 3,289 | 0.325 | 0.235 | 0.000 | 0.130 | 0.476 | 1.000 |
| Control of corruption | 3,181 | -0.001 | 1.006 | -1.723 | -0.757 | 0.642 | 2.470 |
| Governm. effectiveness | 3,176 | 0.057 | 0.959 | -2.078 | -0.672 | 0.708 | 2.437 |
| Regulatory quality | 3,176 | 0.088 | 0.937 | -2.364 | -0.577 | 0.820 | 2.261 |
| Rule of law | 3,196 | -0.003 | 0.977 | -2.322 | -0.756 | 0.734 | 2.100 |
| Voice and accountability | 3,190 | -0.010 | 0.943 | -2.233 | -0.762 | 0.825 | 1.801 |
| Conflict | 3,696 | 0.136 | 0.343 | 0 | 0 | 0 | 1 |

## Supplementary Table 2: Replication of all regressions, excluding investment in hydroelectricity

| **Influence on level of democracy** | | | | | | | |
| --- | --- | --- | --- | --- | --- | --- | --- |
|  |  | | | | | | |
|  | Participatory democracy index (Varieties of Democracy) | | | | | | |
|  | (1) | (2) | (3) | (4) | (5) | (6) | (7) |
|  | | | | | | | |
| RE inv. HHI | 0.009 | 0.008 | 0.008 | 0.006 | 0.004 | 0.005 | 0.0002 |
|  | (0.007) | (0.007) | (0.006) | (0.007) | (0.006) | (0.006) | (0.006) |
| GDP/capita |  | -0.00000^*^ |  |  |  | -0.00000^***^ | -0.00000^***^ |
|  |  | (0.00000) |  |  |  | (0.00000) | (0.00000) |
| Rule of law |  |  | 0.066^***^ |  |  | 0.072^***^ | 0.071^***^ |
|  |  |  | (0.011) |  |  | (0.012) | (0.012) |
| Pol.stab |  |  | 0.011 |  |  | 0.010 | 0.010 |
|  |  |  | (0.008) |  |  | (0.007) | (0.007) |
| Fin.dev.index |  |  |  | -0.162^***^ |  | -0.166^***^ | -0.164^***^ |
|  |  |  |  | (0.025) |  | (0.025) | (0.024) |
| Conflict |  |  |  |  | -0.043^**^ |  | -0.021 |
|  |  |  |  |  | (0.022) |  | (0.018) |
| RE inv. HHI _conflict |  |  |  |  | 0.031 |  | 0.026 |
|  |  |  |  |  | (0.019) |  | (0.016) |
|  | | | | | | | |
| Observations | 807 | 798 | 779 | 788 | 807 | 760 | 760 |
| R^2^ | 0.003 | 0.006 | 0.068 | 0.030 | 0.016 | 0.097 | 0.102 |
| F Statistic | 2.046 (df = 1; 656) | 1.867 (df = 2; 647) | 15.379^***^ (df = 3; 631) | 9.775^***^ (df = 2; 642) | 3.453^**^ (df = 3; 654) | 13.212^***^ (df = 5; 616) | 9.921^***^ (df = 7; 614) |
|  | | | | | | | |
| *Note:* | *p<0.1;* ***p<0.05;*** p<0.01 | | | | | | |

| **Influences on level of economic development** | | | | | | | |
| --- | --- | --- | --- | --- | --- | --- | --- |
|  |  | | | | | | |
|  | Economic development (GDP per capita, Worldbank) | | | | | | |
|  | (1) | (2) | (3) | (4) | (5) | (6) | (7) |
|  | | | | | | | |
| RE inv. total | 0.045^***^ | 0.039^**^ | 0.044^***^ | 0.101^***^ | 0.049^***^ | 0.085^***^ | 0.103^***^ |
|  | (0.017) | (0.015) | (0.017) | (0.033) | (0.018) | (0.031) | (0.037) |
| Regulatory quality |  | 4,376.992^***^ |  |  |  | 4,756.998^***^ | 4,866.882^***^ |
|  |  | (1,167.225) |  |  |  | (1,291.313) | (1,289.730) |
| Pol.stab |  | -572.320 |  |  |  | -680.839 | -517.915 |
|  |  | (614.054) |  |  |  | (614.703) | (662.857) |
| Fin.dev.index |  |  | -5,342.864 |  |  | -4,418.279 | -4,279.935 |
|  |  |  | (5,217.265) |  |  | (5,247.776) | (5,244.607) |
| Fuel.exports.share |  |  |  | 21.800 |  | 17.727 | 23.810 |
|  |  |  |  | (38.749) |  | (38.997) | (40.160) |
| Conflict |  |  |  |  | 1,106.307^*^ |  | 1,298.401^*^ |
|  |  |  |  |  | (646.125) |  | (749.334) |
| RE inv. total  _conflict |  |  |  |  | -0.081^**^ |  | -0.140^***^ |
|  |  |  |  |  | (0.036) |  | (0.051) |
|  | | | | | | | |
| Observations | 802 | 773 | 789 | 761 | 802 | 724 | 724 |
| R^2^ | 0.009 | 0.032 | 0.011 | 0.020 | 0.012 | 0.044 | 0.050 |
| F Statistic | 5.772^**^ (df = 1; 649) | 6.915^***^ (df = 3; 624) | 3.683^**^ (df = 2; 642) | 6.174^***^ (df = 2; 620) | 2.591^*^ (df = 3; 647) | 5.404^***^ (df = 5; 589) | 4.430^***^ (df = 7; 587) |
|  | | | | | | | |
| *Note:* | *p<0.1;* ***p<0.05;*** p<0.01 | | | | | | |

| **Influence on levels of human development** | | | | | | | |
| --- | --- | --- | --- | --- | --- | --- | --- |
|  | | | | | | | |
|  | Human Development Index (UNDP) | | | | | | |
|  | (1) | (2) | (3) | (4) | (5) | (6) | (7) |
|  | | | | | | | |
| RE inv. HHI | 0.002 | 0.0002 | 0.002 | 0.002 | 0.002 | 0.004^*^ | 0.004^**^ |
|  | (0.002) | (0.002) | (0.002) | (0.002) | (0.002) | (0.002) | (0.002) |
| Regulatory quality |  | 0.008^**^ |  |  | 0.005 |  | 0.006^*^ |
|  |  | (0.003) |  |  | (0.003) |  | (0.003) |
| Pol.stab |  | 0.003^*^ |  |  | 0.004^**^ |  | 0.004^*^ |
|  |  | (0.002) |  |  | (0.002) |  | (0.002) |
| Fin.dev.index |  |  | 0.071^***^ |  | 0.073^***^ |  | 0.073^***^ |
|  |  |  | (0.013) |  | (0.016) |  | (0.015) |
| Fuel.exports.share |  |  |  | -0.0002^**^ | -0.0002^**^ |  | -0.0003^**^ |
|  |  |  |  | (0.0001) | (0.0001) |  | (0.0001) |
| Conflict |  |  |  |  |  | 0.007 | 0.009^*^ |
|  |  |  |  |  |  | (0.005) | (0.006) |
| RE inv. HHI _conflict |  |  |  |  |  | -0.014^***^ | -0.015^***^ |
|  |  |  |  |  |  | (0.005) | (0.005) |
|  | | | | | | | |
| Observations | 724 | 696 | 717 | 694 | 659 | 724 | 659 |
| R^2^ | 0.001 | 0.025 | 0.073 | 0.009 | 0.104 | 0.022 | 0.124 |
| F Statistic | 0.803 (df = 1; 583) | 4.774^***^ (df = 3; 558) | 22.919^***^ (df = 2; 579) | 2.406^*^ (df = 2; 560) | 12.322^***^ (df = 5; 531) | 4.306^***^ (df = 3; 581) | 10.671^***^ (df = 7; 529) |
|  | | | | | | | |
| *Note:* | *p<0.1;* ***p<0.05;*** p<0.01 | | | | | | |

| **Influence on political stability** | | | | | |
| --- | --- | --- | --- | --- | --- |
|  | | | | | |
|  | Political stability and absence of violence | | | | |
|  | (1) | (2) | (3) | (4) | (5) |
|  | | | | | |
| RE inv. HHI | 0.049 | 0.054 | 0.033 | 0.044 | 0.031 |
|  | (0.036) | (0.036) | (0.035) | (0.036) | (0.034) |
| Fin.dev.index |  | 0.442^*^ |  |  | 0.136 |
|  |  | (0.261) |  |  | (0.298) |
| Fuel.exports.share |  |  | 0.004 |  | 0.005^*^ |
|  |  |  | (0.003) |  | (0.003) |
| HDI |  |  |  | 1.668^**^ | 2.254^***^ |
|  |  |  |  | (0.750) | (0.841) |
|  | | | | | |
| Observations | 788 | 764 | 737 | 772 | 726 |
| R^2^ | 0.003 | 0.009 | 0.006 | 0.009 | 0.020 |
| F Statistic | 1.968 (df = 1; 634) | 2.727^*^ (df = 2; 619) | 1.917 (df = 2; 597) | 2.735^*^ (df = 2; 623) | 3.045^**^ (df = 4; 589) |
|  | | | | | |
| *Note:* | *p<0.1;* ***p<0.05;*** p<0.01 | | | | |

## Supplementary Table 3: Replications of H1 regressions with different democracy indicators

|  | ***Liberal democracy index (Varieties of Democracy)*** | | | | | | |
| --- | --- | --- | --- | --- | --- | --- | --- |
|  | (1) | (2) | (3) | (4) | (5) | (6) | (7) |
|  | | | | | | | |
| RE inv. HHI | 0.010 | 0.010 | 0.0003 | 0.009 | 0.003 | -0.002 | -0.009 |
|  | (0.008) | (0.008) | (0.008) | (0.008) | (0.007) | (0.007) | (0.007) |
| GDP/capita |  | -0.00000 |  |  |  | -0.00000 | -0.00000 |
|  |  | (0.00000) |  |  |  | (0.00000) | (0.00000) |
| Rule of law |  |  | 0.094^***^ |  |  | 0.101^***^ | 0.099^***^ |
|  |  |  | (0.012) |  |  | (0.012) | (0.012) |
| Pol.stab |  |  | 0.021^***^ |  |  | 0.022^***^ | 0.022^***^ |
|  |  |  | (0.006) |  |  | (0.006) | (0.007) |
| Fin.dev.index |  |  |  | -0.148^***^ |  | -0.217^***^ | -0.220^***^ |
|  |  |  |  | (0.032) |  | (0.035) | (0.036) |
| Conflict |  |  |  |  | -0.050^**^ |  | -0.029 |
|  |  |  |  |  | (0.024) |  | (0.020) |
| RE inv. HHI _conflict |  |  |  |  | 0.040^*^ |  | 0.034^*^ |
|  |  |  |  |  | (0.023) |  | (0.020) |
|  | | | | | | | |
| Observations | 1,053 | 1,043 | 994 | 1,026 | 1,053 | 968 | 968 |
| R^2^ | 0.002 | 0.003 | 0.093 | 0.014 | 0.013 | 0.116 | 0.121 |
| F Statistic | 1.954 (df = 1; 883) | 1.310 (df = 2; 873) | 28.315^***^ (df = 3; 826) | 6.104^***^ (df = 2; 861) | 3.809^***^ (df = 3; 881) | 21.160^***^ (df = 5; 804) | 15.829^***^ (df = 7; 802) |
|  | | | | | | | |
| *Note:* | *p<0.1;* ***p<0.05;*** p<0.01 | | | | | | |

|  | ***Polity IV index*** | | | | | | |
| --- | --- | --- | --- | --- | --- | --- | --- |
|  | (1) | (2) | (3) | (4) | (5) | (6) | (7) |
|  | | | | | | | |
| RE inv. HHI | 0.358 | 0.250 | 0.180 | 0.288 | 0.233 | 0.034 | -0.137 |
|  | (0.220) | (0.214) | (0.214) | (0.212) | (0.171) | (0.196) | (0.162) |
| GDP/capita |  | -0.0001^***^ |  |  |  | -0.00005^***^ | -0.00005^***^ |
|  |  | (0.00001) |  |  |  | (0.00001) | (0.00001) |
| Rule of law |  |  | 2.447^***^ |  |  | 2.750^***^ | 2.721^***^ |
|  |  |  | (0.403) |  |  | (0.437) | (0.435) |
| Pol.stab |  |  | 0.271 |  |  | 0.319 | 0.315 |
|  |  |  | (0.249) |  |  | (0.249) | (0.248) |
| Fin.dev.index |  |  |  | -9.185^***^ |  | -10.126^***^ | -10.196^***^ |
|  |  |  |  | (1.112) |  | (1.431) | (1.448) |
| Conflict |  |  |  |  | -0.723 |  | -0.720 |
|  |  |  |  |  | (0.772) |  | (0.626) |
| RE inv. HHI _conflict |  |  |  |  | 0.652 |  | 0.840 |
|  |  |  |  |  | (0.778) |  | (0.675) |
|  | | | | | | | |
| Observations | 963 | 954 | 904 | 940 | 963 | 882 | 882 |
| R^2^ | 0.003 | 0.024 | 0.058 | 0.058 | 0.006 | 0.131 | 0.135 |
| F Statistic | 2.557 (df = 1; 803) | 9.852^***^ (df = 2; 794) | 15.416^***^ (df = 3; 746) | 24.208^***^ (df = 2; 784) | 1.570 (df = 3; 801) | 21.904^***^ (df = 5; 727) | 16.105^***^ (df = 7; 725) |
|  | | | | | | | |
| *Note:* | *p<0.1;* ***p<0.05;*** p<0.01 | | | | | | |
